# *TP53* Arg72Pro, mortality after cancer, and all-cause mortality in 105,200 individuals

**DOI:** 10.1038/s41598-017-00427-x

**Published:** 2017-03-23

**Authors:** Jakob B. Kodal, Signe Vedel-Krogh, Camilla J. Kobylecki, Børge G. Nordestgaard, Stig E. Bojesen

**Affiliations:** 1 Department of Clinical Biochemistry, Herlev and Gentofte Hospital, Copenhagen University Hospital, DK-2730 Herlev, Denmark; 2 Faculty of Health and Medical Sciences, University of Copenhagen, Copenhagen, Denmark; 3 The Copenhagen General Population Study, Herlev and Gentofte Hospital, Copenhagen University Hospital, Herlev, Denmark

## Abstract

Rs1042522 (Arg72Pro) is a functional polymorphism of *TP53*. Pro72 has been associated with lower all-cause mortality and lower mortality after cancer. We hypothesized that *TP53* Pro72 is associated with lower mortality after cancer, lower all-cause mortality, and with increased cancer incidence in the general population in a contemporary cohort. We genotyped 105,200 individuals aged 20–100 years from the Copenhagen General Population Study, recruited in 2003–2013, and followed them in Danish health registries. During follow-up 5,531 individuals died and 5,849 developed cancer. Hazard ratios for mortality after cancer were 1.03 (95% confidence interval:0.93–1.15) for Arg/Pro and 0.96 (95% CI:0.79–1.18) for Pro/Pro versus Arg/Arg. Hazard ratios for all-cause mortality were 0.99 (95% CI:0.93–1.04) for Arg/Pro and 1.09 (95% CI:0.98–1.21) for Pro/Pro versus Arg/Arg. Risk of cancer specific mortality, cardiovascular mortality, and respiratory mortality were not associated with Arg72Pro genotype overall; however, in exploratory subgroup analyses, genotype-associated risks of malignant melanoma and diabetes were altered. Considering multiple comparisons the latter findings may represent play of chance. The *TP53* Arg72Pro genotype was not associated with mortality after cancer, all-cause mortality, or cancer incidence in the general population in a contemporary cohort. Our main conclusion is therefore a lack of reproducing an effect of *TP53* Arg72Pro genotype on mortality.

## Introduction

The tumor suppressor protein p53 regulates the response to oncogene activation and cellular stress. It functions as a transcription factor, hereby regulating apoptotic genes such as *BAX* and *PERP* as well as cell cycle arrest genes such as *CDKN1A*. Also, through interaction with other proteins, such as members of the BCL-2 family, p53 can initiate mitochondrial apoptotic events^1^. Somatic mutations in the *TP53* gene, encoding the p53 protein, are present in approximately half of all human cancers^2^, and it is the most frequently mutated gene in cancers^3^. In mice, enhanced p53 activity is associated with a higher resistance to spontaneous tumor development, but also with shorter lifespan and an earlier onset of some, but not all phenotypes associated with aging^4, 5^.

The germline single nucleotide polymorphism rs1042522 (Arg72Pro) is an arginine (Arg) to proline (Pro) substitution in codon 72, and the two p53 variants differ in how they influence downstream cellular processes; the Arg72 variant induces apoptosis five times better than the Pro72 variant, likely through a greater ability of the Arg72 variant to localize to the mitochondria^6^ and to increase transcription of a number of pro-apoptotic p53-regulated genes^7^.

The Arg72Pro polymorphism has been extensively studied as a risk factor for development of cancer in candidate gene studies^8–13^; however, candidate gene studies can be of low quality and may suffer from a variety of reporting, analysis, and genotyping biases^14–16^. Fewer studies have examined the association of the Arg72Pro polymorphism with all-cause mortality. At present, only two cohort studies recruiting individuals in 1987–1999^17^ and 1991–1994^18^ have addressed this question and both found a lower mortality among homozygotes for the Pro72 variant^17, 18^. Collectively, results from these studies support that the Arg72Pro influences all-cause mortality and mortality after cancer. We hypothesized that *TP53* Pro72 is associated with lower mortality after cancer, lower all-cause mortality, and with increased cancer incidence in the general population in a contemporary cohort.

## Methods

### Study population

We included 105,200 individuals aged 20–100 years from the Copenhagen General Population Study, a prospective population-based cohort study initiated in 2003 with ongoing enrolment. All Danes are given a unique number for identification at birth or immigration and are registered in the national Danish Civil Registration System. This unique identification number can then be used to follow the participants through the national registers with complete follow-up^19^. Danish inhabitants of suburban Copenhagen areas were invited using the national Danish Civil Registration System. In order to minimize the risk of population stratification, only individuals of Danish descent were included. All participants filled out an extensive questionnaire on life-styles and health, which was reviewed by the participant together with an investigator at the day of study attendance, had a physical examination done, and had blood samples taken for biochemical analysis and DNA extraction. The study was approved by Herlev and Gentofte Hospital, a Danish ethics committee, and was conducted according to the Declaration of Helsinki. Written informed consent was obtained from all participants.

### Endpoints

We followed all individuals until death (n = 5,531), emigration (n = 392) or November 14, 2014, whichever came first. No individuals were lost to follow-up. Date of death or emigration was obtained from the national Danish Civil Registration System. Causes of death were obtained from the national Danish Causes of Death Registry. Records rank main and contributing causes of death as reported by general practitioner, hospital doctor, or by a physician in a forensic or pathology department, using WHO’s tenth International Classification of Diseases (ICD-10)^20^. Cause of death was defined as cancer specific, cardiovascular, or respiratory if the highest ranked cause of death was a diagnosis of cancer (ICD-10 C00-C97), cardiovascular disease (ICD-10 I00-I99), or respiratory disease (ICD-10 J00-J99), respectively. If the highest ranked cause of death was none of the above, but a diagnosis was available, then cause of death was categorized as “other”. Information on the diagnosis of cancer until December 31, 2012 was obtained from the national Danish Cancer Registry, which was established in 1943. Since 1987, it has been compulsory for all physicians by law to report cancer diagnosis to the national Danish Cancer Registry^21^. Cancer diagnoses were assembled into 8 groups to maximize statistical power. Gastrointestinal cancer included pharynx, esophagus, stomach, colon, rectum, liver, and pancreas cancer. Respiratory cancer included larynx and lung cancer. Urologic cancer included bladder and kidney cancer. Hematologic cancer included non-Hodgkin lymphoma, Hodgkin disease, multiple myeloma, and leukaemia. Male cancer included prostate and testis cancer. Female cancer included female breast, cervix uteri, corpus uteri, and ovary cancer. Other cancers included brain and central nervous system, thyroid cancer, sarcomas, and cancer of unknown primary origin.

### Genotypes

We extracted DNA from leukocytes in peripheral blood using Qiagen blood kit for DNA extraction. Genotyping of 105,200 individuals for the *TP53* rs1042522 (Arg72Pro) variant was done with a TaqMan-based assay (Applied Biosystems). Sequence of primers and probes are available upon request. Samples with failed genotyping were attempted to be genotyped again, and a second time if failed. Thereby, 99.9% of available samples were genotyped. Control samples for correct genotyping were obtained from the Copenhagen City Heart Study, previously genotyped using a different technique^18, 22^.

### Covariates

Information on covariates was derived from the questionnaire, physical examination, and blood measurements recorded at the day of attendance. We defined physical inactivity as being completely physically inactive or physical active for a maximum of two hours per week. Completed higher education was defined as having a higher education of 3 years or longer. High annual household income was defined as more than 600,000 DKK per year, equivalent to 80,625 euros. Weekly alcohol intake was self-reported number of units, converted to g/week (1 unit ≈12 g). Cumulative smoking in pack-years was calculated as the cumulated amount of tobacco smoked by the individual, divided by the equivalent of smoking 20 cigarettes a day for an entire year. Diabetes was defined as self-reported diabetes, use of insulin, use of oral antidiabetics, nonfasting blood glucose >11 mmol/L (198 mg/dL) at the day of examination, and/or a diagnosis of diabetes in the national Danish Patient Registry before the date of baseline examination. Body mass index was calculated as measured weight in kilograms divided by measured height in meters squared. Plasma cholesterol was measured using a standard hospital assay.

### Statistical analysis

We used Stata/SE 13.1. We used Cuzick’s nonparametric test for trend to test for associations of genotype with baseline characteristics. The Hardy-Weinberg Equilibrium hypothesis was tested using Pearson’s χ^2^ test.

Hazard ratios for mortality after cancer were calculated using Cox proportional hazards regression analysis adjusted for sex and age (as underlying time scale), since Arg72Pro was not associated with any of the measured potential confounders included in baseline characteristics in Table 1. We included all individuals who received a cancer diagnosis after the examination date and before December 31, 2012, the date where cancer follow-up ended. In these analyses, entry was defined as the day of cancer diagnosis.

**Table 1:** Baseline characteristics according to *TP53* Arg72Pro genotype in individuals in the general population.

Characteristic	Arg72Pro genotype			
	Arg/Arg	Arg/Pro	Pro/Pro	p-value
Individuals	56,559 (54)	41,233 (39)	7,408 (7)	
Male	25,464 (45)	18,475 (45)	3,376 (46)	0.86
Age, years	58 (48–67)	58 (48–67)	58 (48–67)	0.78
Physically inactive	3,506 (6)	2,508 (6)	480 (6)	0.87
Completed higher education	25,688 (45)	18,749 (45)	3,383 (46)	0.71
High annual household income	24,387 (43)	17,814 (43)	3,265 (44)	0.23
Alcohol, g/wk	96 (48–180)	96 (48–180)	96 (48–180)	0.10
Current or former smoker	33,031 (58)	24,085 (58)	4,253 (57)	0.29
Total tobacco consumption, pack-years^c^	15 (6–30)	15 (6–30)	15 (6–30)	0.51
Diabetes mellitus	2,692 (5)	2,058 (5)	377 (5)	0.07
Body mass index, kg/m^2^	25.6 (23.2–28.5)	25.5 (23.2–28.4)	25.5 (23.2–28.4)	0.04^NS^
Systolic blood pressure, mmHg	140 (126–155)	140 (126–155)	140 (126–155)	0.94
Plasma cholesterol, mmol/liter	5.5 (4.8–6.3)	5.5 (4.9–6.3)	5.5 (4.9–6.3)	0.11

Data are expressed as number and percentage for categorical values and median and interquartile range for continuous values. Total tobacco consumption was calculated for current and former smokers only. P-values were calculated using Cuzick’s test for trend, and the nominal p-values are shown without prior adjustment for multiple comparisons. ^NS^Insignificant after Bonferroni correction for 12 multiple tests. Required p-value = 0.004 (=0.05/12).

For all-cause mortality, all individuals were included. Hazard ratios were calculated using Cox proportional hazards regression analysis adjusted for sex and age (as time scale), with entry as the day of examination. For risk of cancer, we used Cox proportional hazards regression analysis adjusted for sex and age (as time scale), with entry at birth or at the start of the Danish cancer registry if individuals were born before January 1, 1943.

Proportional hazards over time were assessed based on Schoenfeld residuals. No major violations of the proportional hazard assumption were noted.

Interactions between Arg72Pro genotype and age, sex, smoking status, and body mass index, covariates that could influence the association between genotype and all-cause mortality, were tested using the Likelihood-ratio test by introducing a two-factor interaction term in a model including both factors. Estimates with confidence intervals from other studies were compared with our results using the Bland-Altman test^23^. A meta-analysis was conducted using the method of DerSimonian and Laird, with the estimate of heterogeneity taken from the Mantel-Haenszel model. This method takes the number of individuals per study into consideration.

P-values were calculated from two-tailed analyses and nominal values are shown throughout the paper. For each display item, several exploratory analyses were performed, lowering the p-value required for significance below the conventional 0.05. Therefore, we note the significance cut-off a.m. Bonferroni in all the figure and table legends.

## Results

Among the 105,200 individuals from the Copenhagen General Population Study, rs1042522 (Arg72Pro) genotype frequencies were 54% for Arg/Arg homozygotes, 39% for Arg/Pro heterozygotes, and 7% for Pro/Pro homozygotes, not differing from Hardy-Weinberg equilibrium (p-value = 0.39). Baseline characteristics did not differ across Arg72Pro genotypes (Table 1), nor after stratification according to sex (Table 2). Median follow-up was 5.6 years (interquartile range: 3.0–8.1 years).

**Table 2:** Incidence of sex-specific cancers and distribution of baseline characteristics according to sex and *TP53* Arg72Pro genotype.

Endpoint	N	Events	Arg/Arg	Arg/Pro	Pro/Pro	p for trend
			HR	HR (95% CI)	HR (95% CI)	
Male						
Prostate cancer	39,502	1,652	1.00	0.95 (0.86–1.05)	0.90 (0.74–1.10)	0.21
Testicular cancer	39,502	245	1.00	1.23 (0.95–1.60)	0.87 (0.50–1.51)	0.50
Baseline characteristics, n (%) or median (IQR)						
Age, years	47,315		59 (49–68)	58 (48–68)	58 (48–68)	0.12
Alcohol, g/wk	47,315		144 (72–240)	144 (72–240)	144 (72–240)	0.06
Current or former smoker	47,315		15,847 (62)	11,456 (62)	2,078 (62)	0.42
Total tobacco consumption, pack-years	29,381		20 (9.0–36)	20 (9.0–35)	20 (9.0–35)	0.53
Diabetes mellitus	47,315		1,523 (6)	1,125 (6.1)	193 (5.7)	0.91
Body mass index, kg/m^2^	47,315		26 (24–29)	26 (24–29)	26 (24–29)	0.07
Female						
Breast cancer	48,473	2,793	1.00	1.01 (0.93–1.09)	1.11 (0.96–1.28)	0.34
Cervix cancer	48,473	287	1.00	0.84 (0.66–1.08)	0.93 (0.59–1.48)	0.29
Uterus cancer	48,473	455	1.00	1.01 (0.83–1.23)	0.97 (0.67–1.41)	0.99
Ovary cancer	48,473	264	1.00	0.97 (0.75–1.24)	0.79 (0.46–1.34)	0.45
Baseline characteristics, n (%) or median (IQR)						
Age, years	57,885		57 (48–67)	58 (48–67)	58 (48–67)	0.08
Alcohol, g/wk	57,885		72 (36–132)	72 (36–132)	72 (36–132)	0.67
Current or former smoker	57,885		17,184 (55)	12,629 (55)	2,175 (54)	0.47
Total tobacco consumption, pack-years	31,988		12 (4.5–24)	12 (4.5–25)	12 (4.5–25)	0.69
Diabetes mellitus	57,885		1,169 (3.8)	933 (4.1)	184 (4.6)	0.004^NS^
Body mass index, kg/m^2^	57,885		25 (22–28)	25 (22–28)	25 (22–28)	0.22

Data on baseline characteristics are expressed as number and percentage for categorical values and median and interquartile range (IQR) for continuous values. Hazard ratios for cancer endpoints were adjusted for sex and age. Follow-up started at the day of birth or for individuals born before 1943 at the start of the national Danish Cancer Registry and ended at cancer diagnosis, death, emigration, or December 31, 2012 whichever came first. Total tobacco consumption was calculated for current and former smokers only. P-values were calculated using Cuzick’s test for trend or a Cox regression using Arg72Pro genotype as a continuous variable. The nominal values are shown without prior adjustment for multiple comparisons. ^NS^Insignificant after Bonferroni correction for 18 multiple tests. Required p-value = 0.003 (=0.05/18). HR, Hazard ratio; CI, Confidence interval.

### Mortality after cancer

**Figure 1:**
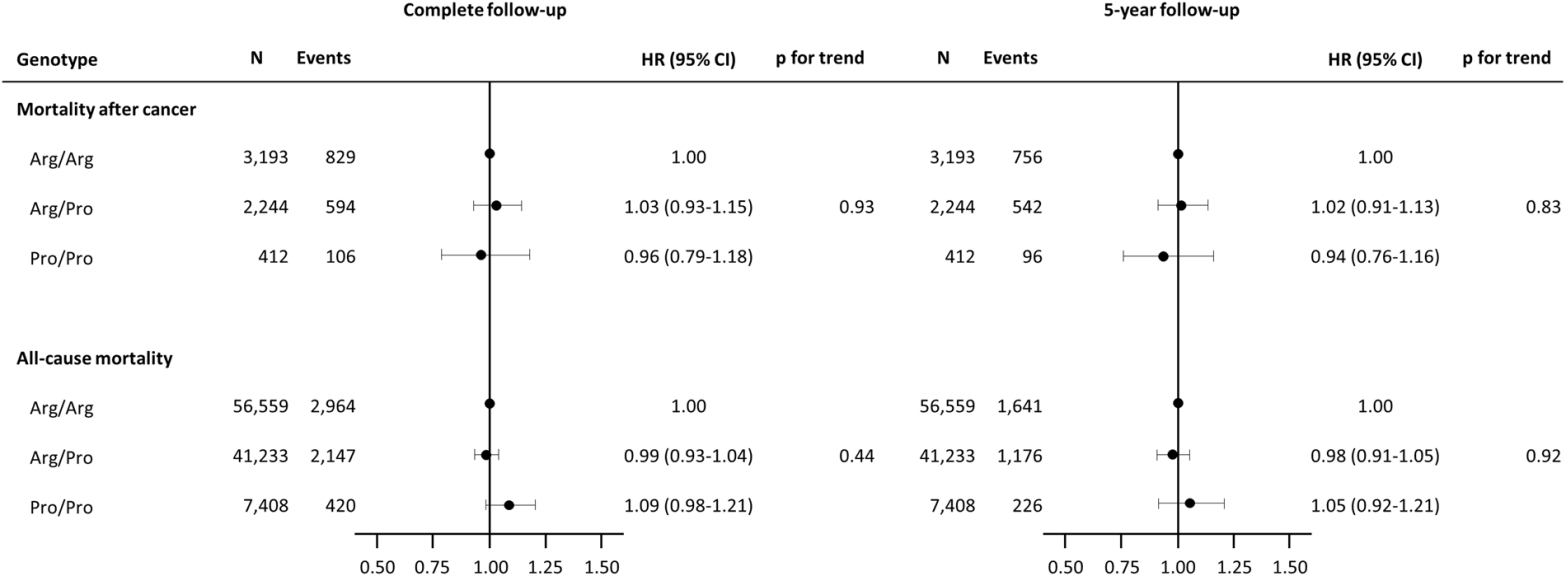
Hazard ratios for mortality after cancer and for all-cause mortality according to *TP53* Arg72Pro genotype in the general population. Hazard ratios were adjusted for sex and age in all analyses. Follow-up started at first cancer diagnosis after baseline (mortality after cancer) or the day of baseline examination (all-cause mortality), and ended on the day of death, emigration or November 14, 2014, whichever came first. In the mortality after cancer analysis with 5 year follow-up, follow-up ended 5 year after cancer diagnosis, death, emigration, or November 14, 2014 whichever came first. In the all-cause mortality analysis with 5 year follow-up, follow-up ended 5 year after baseline examination, death, emigration, or November 14, 2013 whichever came first. CI; Confidence interval. HR; Hazard ratio.

5,849 individuals developed cancer after the baseline examination, and 1,529 of these died during follow-up. The hazard ratio for mortality after cancer was 1.03 (95% confidence interval (CI): 0.93–1.15) for individuals with Arg/Pro and 0.96 (95% CI: 0.79–1.18) for Pro/Pro versus individuals with Arg/Arg (Fig. 1). When restricting follow-up to 5 years after the diagnosis of cancer, results were similar (Fig. 1).

### All-cause mortality

**Figure 2:**
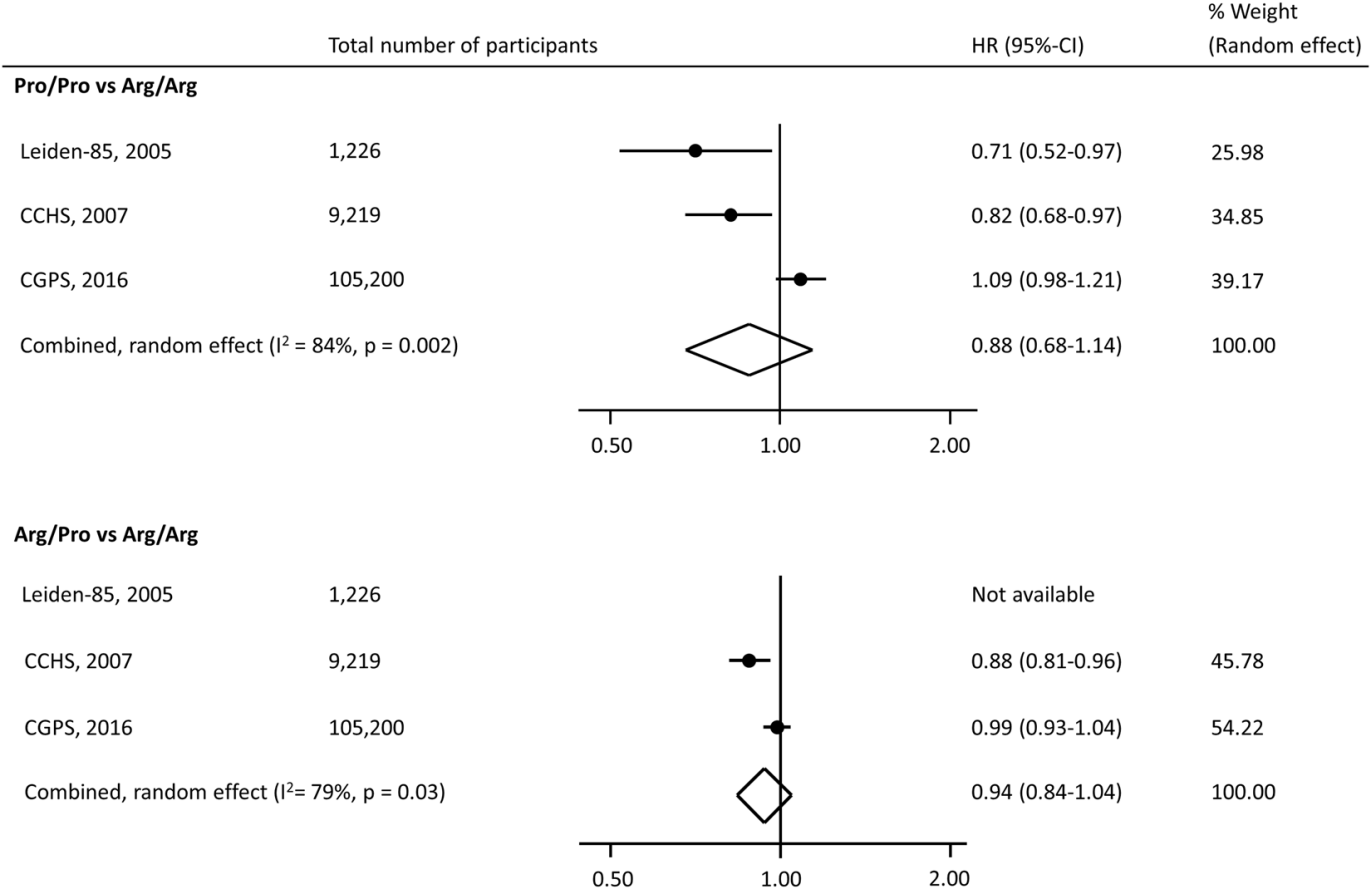
Meta-analysis of hazard ratios for all-cause mortality according to *TP53* Arg72Pro genotype in 3 longitudinal studies. Hazard ratios were adjusted for sex and age. The hazard ratio for Arg/Pro vs. Arg/Arg was not reported in the Leiden 85-plus Study. CCHS; Copenhagen City Heart Study. CGPS; Copenhagen General Population Study. CI; Confidence interval. HR; Hazard ratio. Leiden-85; The Leiden 85-plus Study.

**Figure 3:**
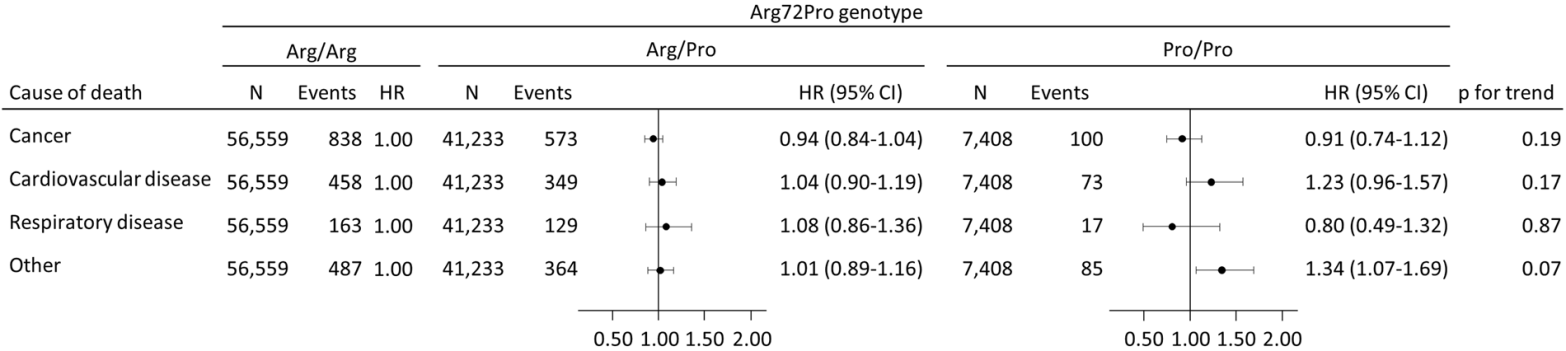
Cause-specific mortality according to *TP53* Arg72Pro genotype in individuals in the general population. Hazard ratios were adjusted for age and sex. Follow-up started at the day of baseline examination and ended at death, emigration or November 14, 2014 whichever came first. Death endpoints were collected from the Danish Civil Registration System. Cause of death was collected from the Danish Register of Causes of Death. The sum of all events is lower than the sum of all-cause mortality events in Fig. 1 since the Danish Register of Causes of Death lags slightly behind the registration of all-cause mortality. Also, the sum of events differs from the number of deaths among cancer diagnosed individuals which are shown in Fig. 1. p-values for trend were calculated using Cox regression with Arg72Pro genotype as a continuous variable. CI; Confidence interval. HR; Hazard ratio.

During follow-up, 5,531 of the 105,200 individuals died. The hazard ratio for all-cause mortality was 0.99 (95% CI: 0.93–1.04) for individuals with Arg/Pro and 1.09 (95% CI: 0.98–1.21) for Pro/Pro versus individuals with Arg/Arg (Fig. 1). Results were similar when restricting analyses to those of 85 years and older (data not shown), as done in a previous study^17^. We found no interaction of the Arg72Pro genotype with age, sex, smoking status, or body mass index on mortality risk (data not shown). In a meta-analysis of the results from this and the published studies on Arg72Pro and all-cause mortality, the estimates were heterogeneous for both the Pro/Pro versus Arg/Arg (I^2^ = 84%, p = 0.002), and for the Arg/Pro versus Arg/Arg (I^2^ = 79%, p = 0.03) analyses. Overall, there was no association between Arg72Pro genotype and all-cause mortality (Fig. 2). When assessing cause-specific mortality, we found no association of the Arg72Pro genotype with cancer specific mortality, cardiovascular specific mortality, or respiratory specific mortality (Fig. 3).

### The Arg72Pro and cancer incidence

**Figure 4:**
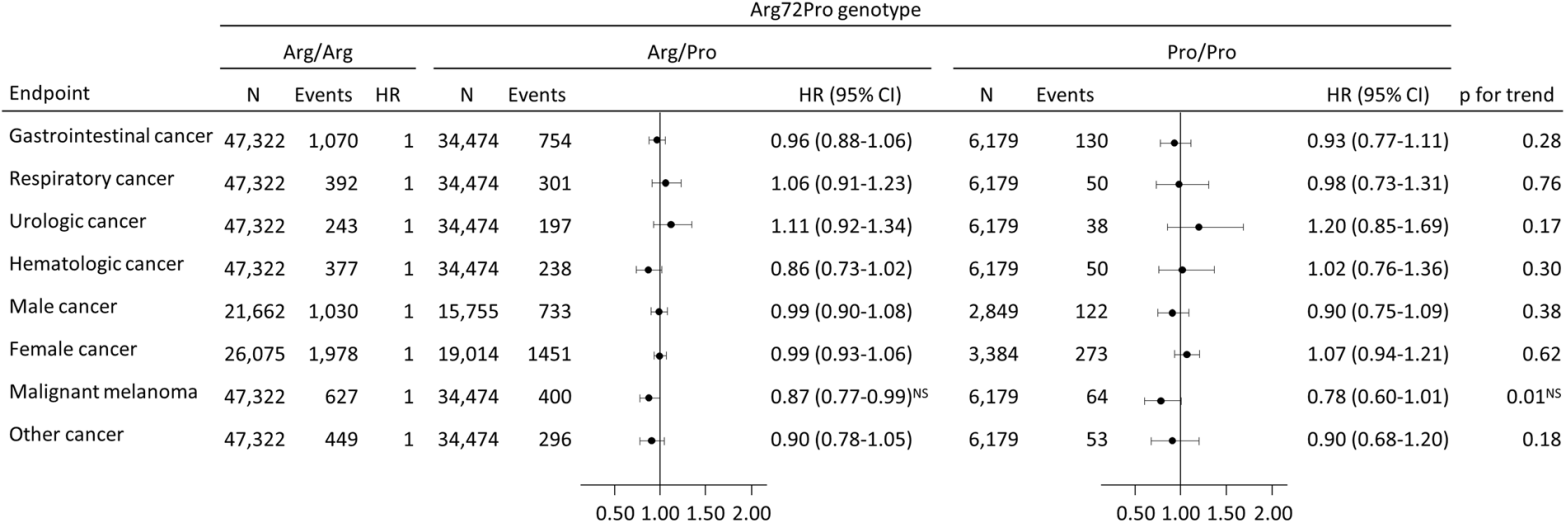
Risk of cancer according to *TP53* Arg72Pro genotype in individuals in the general population. Hazard ratios were adjusted for sex and age. Follow-up started at the day of birth or for individuals born before 1943 at the start of the national Danish Cancer Registry and ended at cancer diagnosis, death, emigration, or December 31, 2012 whichever came the first. p-values for trend were calculated with Cox regression using Arg72Pro genotype as a continuous variable, and the nominal values are shown without prior adjustment for multiple comparisons. ^NS^Insignificant after Bonferroni correction for 8 multiple tests. Required p-value = 0.006 CI; Confidence interval. HR; Hazard ratio.

For malignant melanoma, the hazard ratio was 0.87 (95% CI: 0.77–0.99) for individuals with Arg/Pro and 0.78 (95% CI: 0.60–1.01) for Pro/Pro versus individuals with Arg/Arg with a nominally significant trend for a per-allele effect (p = 0.01); however, this was not significant after Bonferroni correction for eight tests. No other associations between Arg72Pro and cancer incidence were found (Fig. 4), nor between Arg72Pro and incidence of sex-specific cancers (Table 2). Likewise, when stratifying on cancer subtypes there was no association between Arg72Pro and cancer incidence after correcting for multiple comparisons (Supplementary Figure S1).

## Discussion

In this study of 105,200 individuals from the general population recruited in 2003–2013, we found no difference in risk of mortality after cancer, all-cause mortality, or cancer incidence according to Arg72Pro genotype. In exploratory subgroup analyses, genotype associated risks of malignant melanoma and diabetes were altered. Considering multiple comparisons, these findings may represent play of chance.

The association between malignant melanoma and Arg72Pro genotype has been studied by several groups due to a geographical latitude gradient in allele distribution. Among South Africans, 70% of the alleles are Pro72 compared to only 23% among Western Europeans^24^. This has led to the hypothesis that the Pro72 allele is protective against sunlight induced diseases^25^. However, meta-analyses of both non-melanoma skin cancer and malignant melanoma have found no association between Arg72Pro and risk of these cancers^26^.

The Arg72Pro polymorphism has been extensively studied as a risk factor for development of cancer. Several meta-analyses have been conducted in various cancers including cervical, breast, skin, head, and neck cancer^8–13^. However, the results have been inconsistent with reports of potential publication bias^9, 11^ and of genotype misclassification in association studies using tumor tissue as a source of genotyping material^15^. No genome-wide association study has found an association between Arg72Pro and risk of a cancer^27^.

In a recent meta-analysis, the Arg72 allele of Arg72Pro was associated with increased odds of type 2 diabetes^28, 29^. Although, we did find an association between diabetes and genotype at baseline examination in women only, this association did not remain after correction for multiple comparisons, suggesting that the association may represent play of chance.

Two human cohort studies have evaluated the association between all-cause mortality and the Arg72Pro genotype. A Dutch study of individuals aged 85 years or older, the Leiden 85-plus Study including 1,226 individuals, reported a higher survival ratio for Pro/Pro versus Arg/Arg homozygotes but also a higher proportion of death from cancer among Pro/Pro homozygotes^17^. Similarly, a Danish study of 9,219 individuals aged 20–100 years from the Copenhagen City Heart Study reported lower mortality for both Pro/Pro and Pro/Arg versus Arg/Arg^18^. This study also found increased survival after a diagnosis of any cancer for Pro/Pro versus Arg/Arg. These studies suggest a survival benefit for the Pro72 allele, possible through a lower apoptotic-inducing potential^17, 18^. However, our results do not support this.

**Figure 5:**
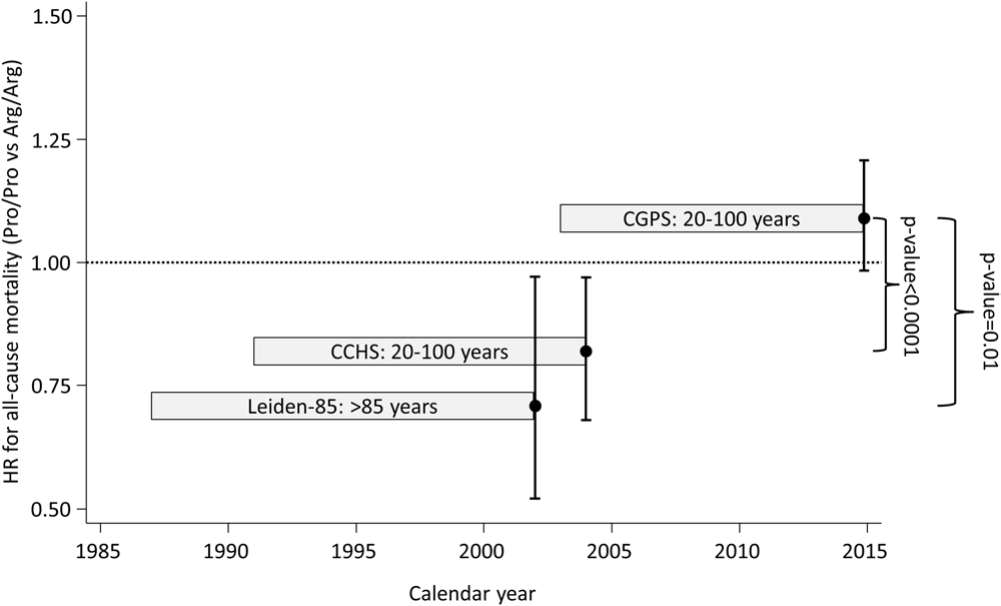
Hazard ratios for all-cause mortality according to *TP53* Arg72Pro genotype (Pro/Pro vs. Arg/Arg) in 3 longitudinal studies. Hazard ratios for death are adjusted for sex and age. Follow-up started at time of recruitment and ended at death, emigration or study termination, whichever came first. The boxes cover the period from the first recruitments until end of follow-up, and hazard ratios with 95% confidence intervals are shown at end of follow-up. CCHS; Copenhagen City Heart Study. CGPS; Copenhagen General Population Study. CI; Confidence interval. HR; Hazard ratio. Leiden-85; The Leiden 85-plus Study.

Our result on all-cause mortality in individuals recruited in 2003–2014, was indeed different from those in the two previous studies recruited in 1987–1999 and 1991–1994 (Figs 2 and 5). The lack of association between Arg72Pro and all-cause mortality observed in the present study could indicate a gene-environment interaction with a change in effect of Pro72 on mortality over calendar time. The disappearing association with reduced all-cause mortality could be caused by more effective prevention or treatments of factors limiting the longevity of Arg72 homozygotes. Additionally, secular trends in mortality as well as in lifestyle and environmental changes could interact with the Arg72Pro genotype and eliminate a survival effect of the Pro72 allele; possible mechanisms may be examined in future studies. Changes in cancer treatment and cancer survival in Denmark during the past twenty years could have led to a diminished effect of Arg72Pro on mortality after cancer^30, 31^. In support of this, a German study with 463 cases and 563 controls, found a possible gene-environment interaction between Arg72Pro and the preventive effect of nonsteroidal anti-inflammatory drugs (NSAID) on risk of colon cancer; Pro72 carriers had a low risk regardless of NSAID use, while Arg72 homozygotes had low risk only when taking NSAID^32^. Recent trends in use of NSAID and aspirin show increasing usage of ibuprofen and low dosage aspirin^33^. This could be one example of an interaction between Arg72Pro genotype and environment, an interaction that may have changed over time and that could influence mortality.

Although the concept of a secular trend between the Arg72Pro polymorphism and all-cause mortality is intriguing, a simpler theoretical explanation may be that many of the studies on *TP53* Arg72Pro and cancer incidence did actually examine the associations with all-cause mortality, but did not publish a lack of association. This in turn could create selective reporting and hence publication bias of the association. Such a possibility together with the effect of genotype misclassification and genotyping errors in candidate gene studies^14, 16^ might explain the discrepancies between previous studies and our findings. As only two other studies have published on the association between the Arg72Pro polymorphism and all-cause mortality^17, 18^, we were not able to statistically evaluate the hypothesis of a publication bias. That said, a meta-analysis combining the results from the two earlier studies with ours showed no association between Arg72Pro genotype and all-cause mortality (Fig. 2). Finally, we cannot exclude the possibility that the earlier findings, or the present, came from play of chance.

This study is by far the largest cohort study examining the effect of Arg72Pro on all-cause mortality and mortality after cancer; however, some limitations should be considered. First, since our study population consists solely of individuals of Danish descent our findings might not apply to other ethnicities; however, this also minimizes the risk of population stratification. Second, we were not able to evaluate gene-gene-interactions such as with the mouse double minute 2 (MDM2) promoter SNP 309 and hence cannot exclude the existence of an interaction. Also, although we include a large number of individuals, 11,316 cancer events, and 5,531 deaths, we cannot rule out an effect of the Arg72Pro too small for our study to detect. Additionally, the use of Bonferroni correction diminishes the likelihood of chance findings (type I errors) due to multiple testing, but also increases the risk of ignoring important association (type II errors). Hence some associations displayed in Table 1, Table 2, Fig. 4 and Supplementary Figure S1 that did not meet the required level of significance after Bonferroni correction but did meet conventional level of significance (p-value < 0.05), could represent true associations. Importantly, however these findings need validation in future studies.

In conclusion, the *TP53* Arg72Pro genotype was not associated with lower mortality after cancer, lower all-cause mortality, or cancer incidence in the general population in a contemporary cohort. Our main conclusion is therefore a lack of reproducing an effect of *TP53* Arg72Pro genotype on mortality.

### Data availability.

Individual participant data from the Copenhagen General population Study are subject to protection from the national Danish Data Protection Agency and we are not allowed to share the data. However, interested researchers can contact members of the Copenhagen General Population Study steering committee (http://binanic.com/CGPS/Contacts.htm) to request limited data access. Additional data are available upon request and requests may be made to the corresponding author.

## Electronic supplementary material

Supplementary information
