# Supplementary material for: TP53 Arg72Pro, mortality after cancer, and all-cause mortality in 105,200 individuals
Source: Sci Rep. 2017 Mar 23;7:336. doi: 10.1038/s41598-017-00427-x (PMC5428447; doi:10.1038/s41598-017-00427-x)
Supplement: Supplementary file 1 — Supplementary information [file 41598_2017_427_MOESM1_ESM.doc]

**Supplementary information**

***TP53* Arg72Pro, mortality after cancer, and all-cause mortality in 105,200 individuals**

Jakob B. Kodal^1,2^, Signe Vedel-Krogh^1,2^, Camilla J. Kobylecki^1,2^, Børge G. Nordestgaard^1,2,3^, Stig E. Bojesen^1,2,3^*

^1^Department of Clinical Biochemistry, Herlev and Gentofte Hospital, Copenhagen University Hospital, DK-2730 Herlev, Denmark
^2^Faculty of Health and Medical Sciences, University of Copenhagen, Denmark
^3^The Copenhagen General Population Study, Herlev and Gentofte Hospital, Copenhagen University Hospital, Denmark


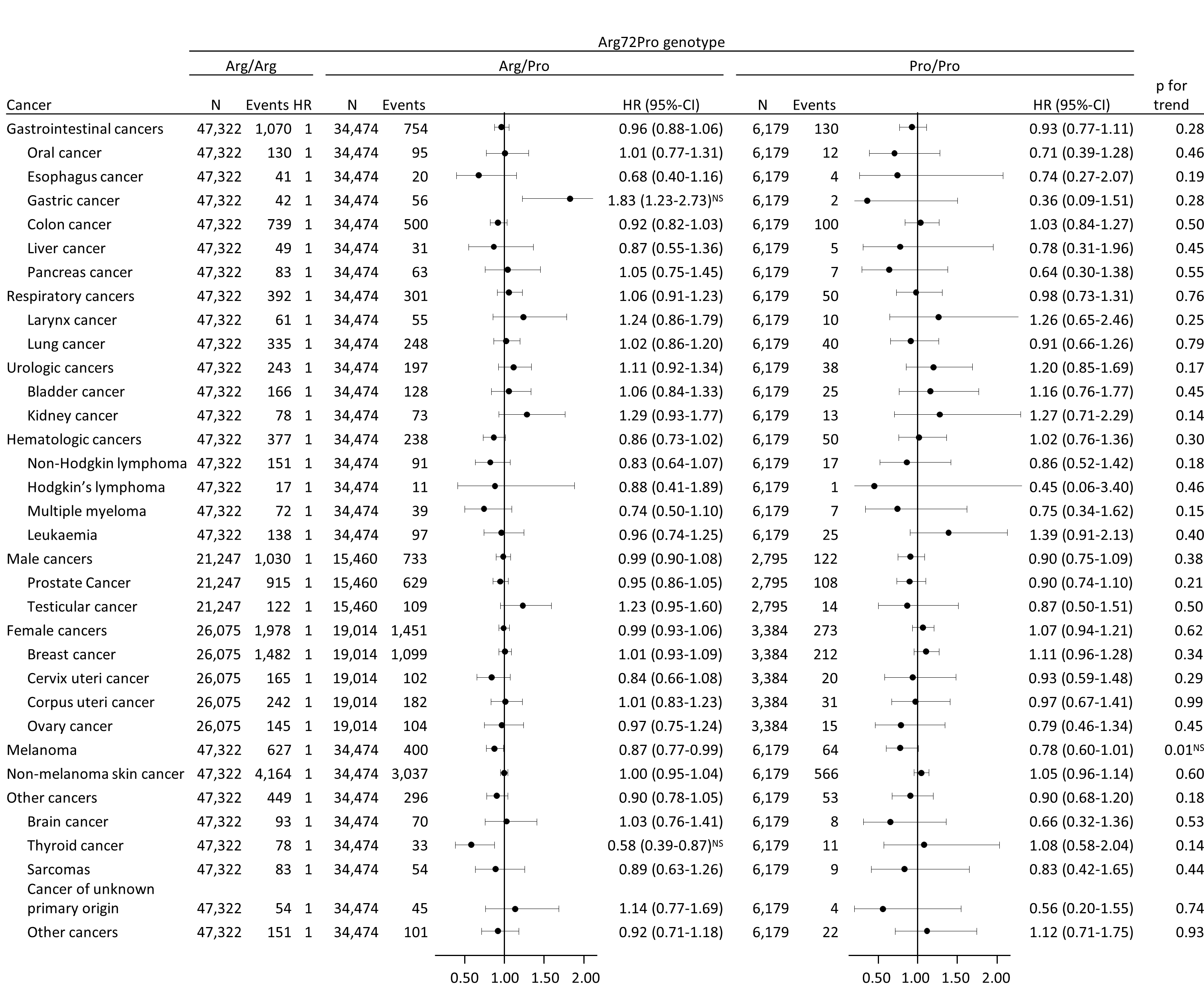


**Supplementary Figure S1: Risk of cancer according to *TP53* Arg72Pro genotype in individuals in the general population.**Hazard ratios were adjusted for sex and age. Follow-up started at the day of birth or for individuals born before 1943 at the start of the national Danish Cancer Registry and ended at cancer diagnosis, death, emigration, or December 31, 2012 whichever came the first. The sum of events in each cancer group differ from the sum of events of the corresponding cancer subgroups as some individuals had more than one cancer per cancer group. P-values were calculated with Cox regression using Arg/Arg as the reference group, and the nominal p-values are shown without prior adjustment for multiple comparisons.
^NS^ Insignificant after Bonferroni correction for 72 multiple tests. Required p-value = 0.0007 (=0.05/72)
HR; Hazard ratio. CI; Confidence interval.
